# How to automatically turn patient experience free-text responses into actionable insights: a natural language programming (NLP) approach

**DOI:** 10.1186/s12911-020-1104-5

**Published:** 2020-05-27

**Authors:** Simone A. Cammel, Marit S. De Vos, Daphne van Soest, Kristina M. Hettne, Fred Boer, Ewout W. Steyerberg, Hileen Boosman

**Affiliations:** 1 IT Department, Leiden University Medical Center, Albinusdreef 2, Postbus 9600, Postzone D-01-P, 2300 RC Leiden, The Netherlands; 2 Department of Surgery, Leiden University Medical Center, Leiden, The Netherlands; 3 Department of Medical Decision Making, Leiden University Medical Center, Leiden, The Netherlands; 4 Department of Quality and Patient Safety, Leiden University Medical Center, Leiden, The Netherlands; 5 Department of Human Genetics, Leiden University Medical Center, Leiden, The Netherlands; 6 Department of Biomedical Data Sciences, Medical Decision Making, Leiden University Medical Center, Leiden, The Netherlands

**Keywords:** Natural language processing, Patient experience analysis, PREM, Text analytics, Data science, Machine learning

## Abstract

**Background:**

Patient experience surveys often include free-text responses. Analysis of these responses is time-consuming and often underutilized. This study examined whether Natural Language Processing (NLP) techniques could provide a data-driven, hospital-independent solution to indicate points for quality improvement.

**Methods:**

This retrospective study used routinely collected patient experience data from two hospitals. A data-driven NLP approach was used. Free-text responses were categorized into topics, subtopics (i.e. n-grams) and labelled with a sentiment score. The indicator ‘impact’, combining sentiment and frequency, was calculated to reveal topics to improve, monitor or celebrate. The topic modelling architecture was tested on data from a second hospital to examine whether the architecture is transferable to another hospital.

**Results:**

A total of 38,664 survey responses from the first hospital resulted in 127 topics and 294 n-grams. The indicator ‘impact’ revealed n-grams to celebrate (15.3%), improve (8.8%), and monitor (16.7%). For hospital 2, a similar percentage of free-text responses could be labelled with a topic and n-grams. Between-hospitals, most topics (69.7%) were similar, but 32.2% of topics for hospital 1 and 29.0% of topics for hospital 2 were unique.

**Conclusions:**

In both hospitals, NLP techniques could be used to categorize patient experience free-text responses into topics, sentiment labels and to define priorities for improvement. The model’s architecture was shown to be hospital-specific as it was able to discover new topics for the second hospital. These methods should be considered for future patient experience analyses to make better use of this valuable source of information.

## Background

Patient experience surveys are a popular means of gathering feedback from patients. Surveys often consist of a combination of closed- and open-ended questions. Closed-ended questions yield quantitative results that can be used to measure patient experiences and derive priorities for improvement [1]. Open-ended questions can complement quantitative measures by providing information on experiences not covered by closed-ended questions and by offering greater detail to help contextualize responses to closed questions. In practice, free-text responses are often underutilized [2]. This may be because analysis of free-text responses requires substantial effort due to the unstructured nature of the responses. Raw free-text data from large scale surveys are therefore not always analysed systematically, risking the loss of potentially valuable insights for hospital improvement.

More sophisticated techniques offer a promising solution to analyse free-text responses efficiently. There is increasing interest in applying Natural Language Processing (NLP) techniques [3] to automatically generate structured data out of texts from large datasets. NLP can discover ‘topics’ occurring in a collection of documents [4] (i.e., topic modelling). Topic modelling was previously applied to categorize patient experience free-text responses into predefined topics [5–12]. These studies used an supervised approach [3] meaning topic names were chosen in advance by the authors (i.e. nursing). This has the advantage of having interpretable topic names, relevant to the authors. A drawback is that manual labelling is time-consuming and could result in an inflexible model that needs to be updated over time. Moreover, manual labelling of data adds a layer of investigator interpretation and is therefore no longer an exact representation of patient feedback, which introduces the risk of human bias. These limitations can be overcome using a data-driven unsupervised topic modelling approach. Research in other industries (e.g., topic modelling on book articles) has shown that unsupervised topic modelling also yields interpretable topics [13, 14]. This approach results in a model capturing patients’ exact words and is updated automatically to capture new topics. The model’s architecture could also be used in other hospitals with the same spoken language.

In addition to studying topics in free-text data, NLP can detect sentiment of a topic, assigning a response with a sentiment score [15] ranging from positive (+ 1) to negative (− 1). Sentiment analysis is a common text classification tool that analyses an incoming message and tells whether the underlying sentiment is positive, negative or neutral. This has previously been used to predict whether patient experiences were positive or negative [5, 7, 16]. To make optimal use of patient feedback, a combination of sentiment and frequency may provide an insightful indicator to represent the impact of an experience. This is important because topics mentioned by many patients are not necessarily topics that evoke the most negative emotions. Other topics may be mentioned infrequently but with very negative sentiment. NLP as a method to process open ended questions has potentially wide-ranging implications such as benchmarking between hospitals on textual data and not only closed survey responses and lets us discover our ‘blind spots’ for quality indicators without having to code textual data manually. Gallan et al. mentioned that ‘A significant percentage of patient who rated their experience with a perfect domain score left a comment categorized as not positive, thus giving rise to stark contrasts between survey scores and comments provided by patients’ [7], indicating that this approach could improve the sensitivity of patient experience surveys.

The aims of this study were to 1) examine whether patient experience free-text responses can be categorized using unsupervised topic modelling; 2) create a combined measure of sentiment and frequency which can be used to indicate priorities for improvement; and 3) assess the transferability of the model’s architecture on patient experience free-text responses from another hospital.

## Methods

### Patients and procedure

This retrospective study used patient experience data from inpatient departments of two hospitals: one university hospital (hospital 1; 882 beds) and one general inner-city hospital (hospital 2; 785 beds). The patient experience survey of these hospitals starts with two open-ended questions: ‘What went remarkably well during your stay?’ (Q1) and ‘What did not go as well during your stay?’ (Q2). The survey questions and responses are fielded in Dutch. All results were literally translated to English. Responses were included if at least one question was answered. The largest sample (hospital 1) was used to develop a modelling architecture. This sample consisted of 23,417 inpatients, discharged between August 2013 and April 2018. The architecture was tested on the second sample (hospital 2) of 2608 inpatients, discharged between October 2017 and October 2018.

### Pre-processing

**Fig. 1:**
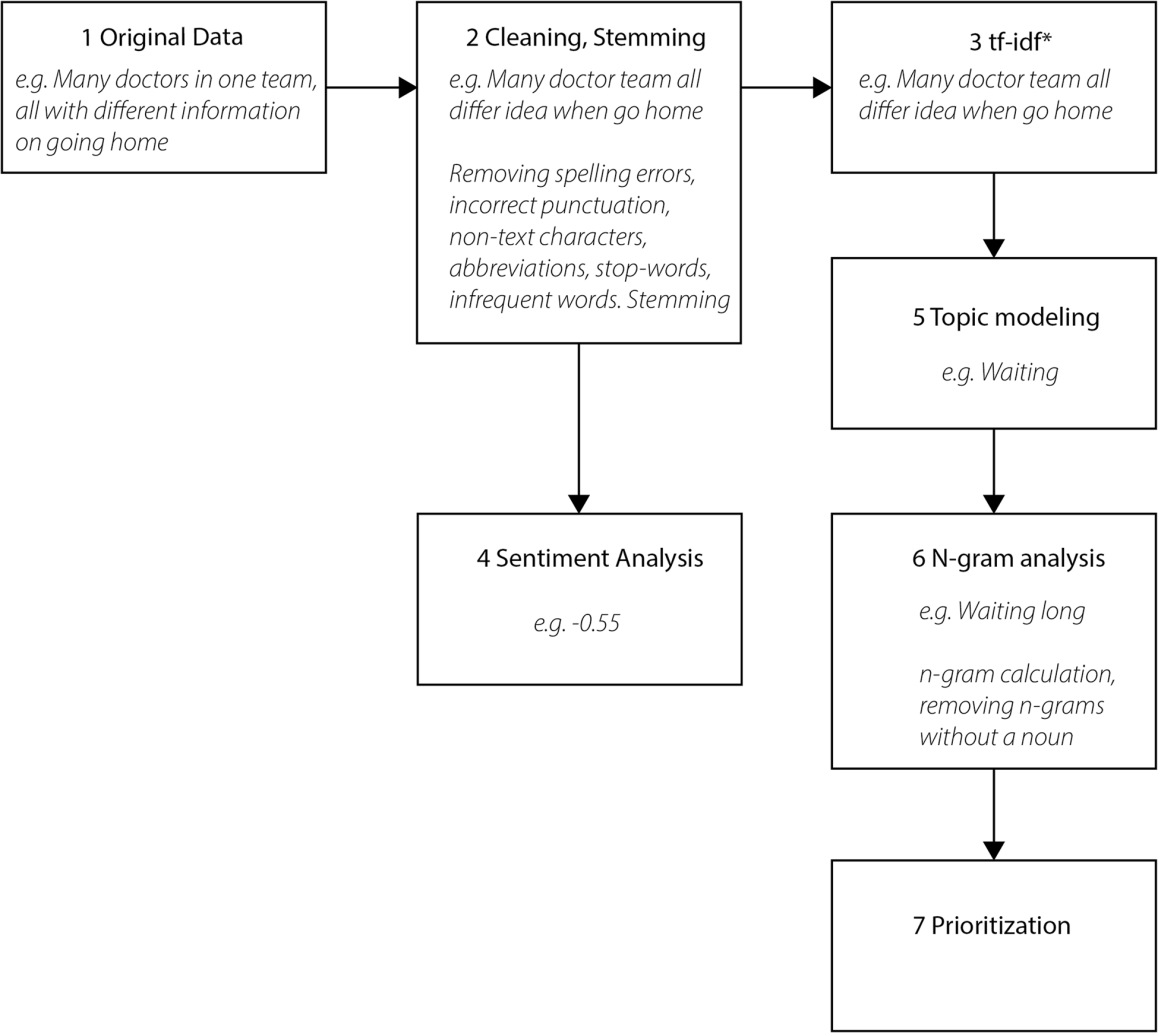
Data flow diagram of data-preprocessing steps used for topic modeling method

Patients’ responses to the open-ended questions were cleaned by automatically removing spelling errors, incorrect punctuation, non-text characters, and abbreviations. For spell-correction, an algorithm based on the Peter Norvig algorithm [17], combined with a Dutch dictionary [18], was used. Frequently occurring (> 100) domain-specific words [19] and abbreviations were added to the dictionary. Large vocabularies are challenging for topic modelling, which aims to reduce the dimensionality (the number of unique words used) of the survey responses to a defined number of topics. Stemming (e.g., studying becomes study), stop-words removal (e.g., the, there) and removal of infrequent words (< 20) (Fig. 1) were used to reduce dimensionality. The resulting unique words together form the ‘corpus’. The corpus was represented term frequency-inverse document frequency (tf-idf) to decrease size of the corpus even further.

Tf-idf discriminates between the importance of terms, meaning frequently occurring word, present in almost every answer (e.g. ‘doctor’) get a lower score indicating lower importance. Frequent words, occurring in a subset of responses (e.g. ‘pain’) get a higher score.

The model was programmed in Python 3.6 [20], including the packages Nltk [21], Numpy [22], scikit-learn [23] and Matplotlib [24].

### Topic modelling

Topic modelling, a technique to identify which topic is discussed in a document or piece of text, was used to categorize patients’ pre-processed responses into topics. These topics were divided into smaller categories, looking at word combinations. The topic model was constructed using non-negative matrix factorization (NMF) [25]. NMF was chosen over popular methods such as Latent Dirichlet Allocation (LDA) because it is more suitable for short texts covering non-mainstream content [26]. All topic models with a varying number of topics (4–100) were analysed on topic coherence. Topic coherence is the extent to which the topic descriptors of a topic are semantically related [27]. Two words that are semantically related have a similar meaning, such as ‘simple’ and ‘easy’. The final topic model was the model with the highest average topic coherence was chosen for the final topic model. Each topic represents a collection of different words contributing to the topic with a weighed H-Factor. For every topic, the topic descriptor with the highest H-Factor was used as a label for that topic.

### N-gram analysis

Topics describe the subject of a text, and can vary in length, ranging from one word to a complete sentence. Single-word topics (topics that only consist of one word) may be too general to guide quality improvement initiatives [28]. N-gram analysis was used to add context to the topics [29] (Additional file 1), because the topic ‘discharge’ may refer to ‘time of discharge’ but also ‘aftercare’. An n-gram is a combination of n adjacent words (E.g. ‘I had pain’ consists of 2-grams ‘I had’ and ‘had pain’). For each topic, a list of the most common 2-, 3-, and 4-g were composed. Only n-grams containing at least one noun were added to enhance interpretability. All patients’ responses assigned to a particular topic were matched against the top 20 n-grams to find the best fitting n-gram with the fuzzy string matching technique [30]. Therefore, each survey answer was not only assigned to a single-word topic, but also to an n-gram label to provide context. The naming of these topics and labels was completely computer-driven.

### Sentiment analysis

Responses were labelled with a sentiment score ranging between − 1.0 (negative) to 1.0 (positive). Sentiment analysis is a text classification tool that can be used to analyse a a piece of text and determine whether the underlying sentiment is positive, negative or neutral. The pattern.nl package [24] provides a list of frequently occurring adjectives (e.g. good, bad) in product reviews. Negations and adverbs of degree (e.g., extremely) impact the sentiment score. Because the data is domain-specific and the list of adjectives and sentiment labels are based on product reviews; some sentiment labels might be incorrect. For example, ‘illness’ is labelled as negative, while in patient experiences ‘disease’ is a frequently occurring word that could be neutral (i.e. I have a disease). Therefore, all frequently occurring words (> 50) with high (> 0.5) or low (<− 0.5) sentiment were manually addressed and if necessary, adjusted.

For validation purposes, the computer-human agreement and inter-rater agreement were examined on a random sample of 200 responses using Fleiss’ Kappa [31]. Three authors individually labelled these as negative, neutral, or positive. These labels were compared to automatically derive sentiment scores, which were also labelled as negative (< 0.0), neutral (0.0–0.1) or positive (> 0.1). These thresholds were decided based on the recommendations of the authors of the pattern.nl package. The statistical analysis was conducted using Python.

### Combining sentiment and frequency of topics

Sentiment and frequency were combined to create a 2 × 2 prioritization matrix. For each n-gram, frequency was plotted against average sentiment. Three areas in the matrix were highlighted:

- *Topics to improve upon*: i.e., frequently mentioned topics (frequency > third quartile) with negative sentiment (< 0.0)
- *Topics to celebrate*: i.e., frequently mentioned topics with positive sentiment (> 0.1)
- Topics to monitor: i.e., frequently mentioned topics with neutral sentiment (0.0–0.1), and all medium frequent topics (median < frequency < third quartile) with negative sentiment

### Prioritization

The n-gram analysis could result in 2000 n-grams (top 20 n-grams for at most 100 topics). Therefore, a prioritization factor was used combining frequency and sentiment to produce a new indicator, referred to as ‘impact’. This is based on the well-known risk calculation combining probability (frequency) and severity of consequences (sentiment) [32]. The formula to calculate impact is shown in Additional file 1.

The result of the impact calculation provides a ranking of n-grams for each category. The top 5 rankings for each category indicate priorities for improvement, monitoring and celebration. Figure 1 shows the flow diagram of the pre-processing steps.

### Transferability of the model

The architecture of the model, not the model itself, was tested on data collected in a second hospital to examine its transferability. Data from hospital 2 were pre-processed and analysed similarly as data from hospital 1. The architecture was applied to the pre-processed data to create a hospital-specific topic model. The topic model of hospital 2 was compared to the topic model of hospital 1. Transferability of the architecture was considered acceptable if the following was demonstrated for hospital 2:

- The number of patient responses that can be assigned to a topic is similar to hospital 1. This is examined using a chi-square goodness-of-fit test
- The model is able to detect unique topics and n-grams which were not present in hospital 1.

## Results

### Description of data

For hospital 1, 20,982 out of 23,417 surveys (89.6%) included a response to Q1 (‘what went remarkably well?’), and 17,682 (75.5%) to Q2 (‘what went less well?’). The original corpus (i.e., list of unique words) consisted of 195,579 words for Q1, and 311,345 for Q2. After pre-processing, this was reduced to 1158 and 1814 words (Table 1). The number of words was dramatically reduced because of the abundant use of stop-words and poor data quality. Removing spelling errors abbreviations reduced the number of unique words to 87% of the original number of words. The rest of the reductions were a result of stemming.

**Table 1:** Data description during preprocessing steps

Hospital	Question^a^	Total no of questions answered	Average no of words per answer	Original corpus size	Corpus size after pre-processing	Optimal no of topics for topic model	No of n-grams
1	Q1: remarkably well	20,982	9.13	195,579	1158	64	165
1	Q2: not as well	17,682	17.85	311,345	1814	63	117
2	Q1: remarkably well	2608	8.33	21,727	216	59	116
2	Q2: not as well	2537	24.93	63,262	628	50	119

^a^ Q1: What went remarkably well during your stay? Q2: What did not go as well during your stay?

### Topic model

NMF topic modelling resulted in 64 topics for Q1 and 63 topics for Q2 (Table 1). Each topic was labelled with the top topic descriptor (Additional file 1). In total, 3435 (16.4%) responses to Q1 and 2529 (14.3%) to Q2 could not be assigned a topic because responses were too short, or consisted of only stop-words.

### N-gram analysis

After assigning n-grams to each survey response, the 17,682 responses to Q1 were reduced to 64 topics and 165 n-grams. The 20,982 replies to Q2 were reduced to 63 topics and 117 n-grams. As an example, the topic ‘surgery’ was divided into four n-grams: ‘anaesthesia eye operation’, ‘waiting room surgery’, ‘insecurity time of surgery’, and ‘hour before surgery’. The n-grams provide additional insight into a topic, showing similar and differing topics (Table 2).

**Table 2:** Top 5 patient experience priorities to celebrate, monitor and improve on for both hospitals

N-gram (literal transllation)	Original (one word topic)	Frequency	Sentiment	Impact	Question	N-gram (translated)	Original (one word) topic	Frequency	Sentiment	Impact	Question
**Hospital 1**						**Hospital 2**					
**Celebrate**						**Celebrate**					
No examples did not go well	No comment	883	0.534	10	2	Very satisfied staff	Satisfied	111	0,437	10	1
Pleasant welcome guidance	Pleasant	322	0.552	10	1	Friendliness doctors staff	Friendliness	64	0,409	5.0	1
Friendliness nursing staff	Friendliness	651	0.314	6.5	1	Complete treatment perfect	Treatment	98	0,279	3.6	1
Staff very kind	Sweet	180	0.519	4.9	1	Acted quickly with expertise	Satisfied	52	0,359	3.1	1
Friendly reception department	Reception	184	0.476	4.3	1	Expertise of staff	Staff	47	0,25	2.34	1
**Monitor**						**Monitor**					
Went wrong once	Mistake	98	−0.580	1.3	2	No emergency department	Emergency department	18	−0.587	5.1	2
Room cold	Cold	155	− 0.428	1.1	2	Going home fast	Speed	25	−0.403	3.3	2
Late communication between staff	Aftercare	148	−0.404	1.0	2	Waiting for results	Waiting	20	−0.345	2.0	2
Discharge unclear took long	Unclear	116	−0.373	0.7	2	Time for patient	Patient	21	−0.256	1.1	2
When could go home	Home	36	−0.638	0.6	2	Took time	Time	20	−0.253	1.0	2
**Improve**						**Improve**					
Long waiting before surgery	Long	393	−0.490	3.7	2	Long waiting in waiting area	Waiting area	45	−0.521	10	2
Leave early from home	Home	222	−0.628	3.5	2	Lower waiting time	Waiting	38	−0.54	9.0	2
Long waiting times	Waiting time	404	−0.287	1.3	2	Only night bad	Night	60	−0.343	5.8	2
Temperature room low	Room	339	−0.295	1.2	2	Communication between departments	Communication	43	−0.351	4.3	2
At times very busy	Busy	159	−0.428	1.2	2	Waiting time to get appointment	Appointment	46	−0.329	4.1	2

N.B. some results can be difficult to interpret due to translation from Dutch to English

### Sentiment analysis

For some frequently occurring words, the sentiment score was adjusted. The negative words ‘disease’, ‘ill’, ‘painful’, ‘nauseous’ were adjusted from their original sentiment score, by increasing the sentiment with 0.3. ‘Help’, ‘remarkable’, ‘waiting’ and ‘complicated’ were adjusted by decreasing the positive sentiment score with 0.3.

The sentiment for Q1, showed a mean of 0.22 (standard deviation (std) 0.31); for Q2 this was − 0.15 (std 0.46). Fleiss’ kappa revealed good agreement between the judgments of the three investigators and the model regarding the sentiment of responses, κ = .810 (95% CI, .761 to .859), *p* < .0005. Raters and the model agreed on the label in 83.3% of the cases. The agreement between investigators also showed good agreement, κ = .849 (95% CI, .779 to .918), p < .0005.

### Sentiment and frequency combined

**Fig. 2:**
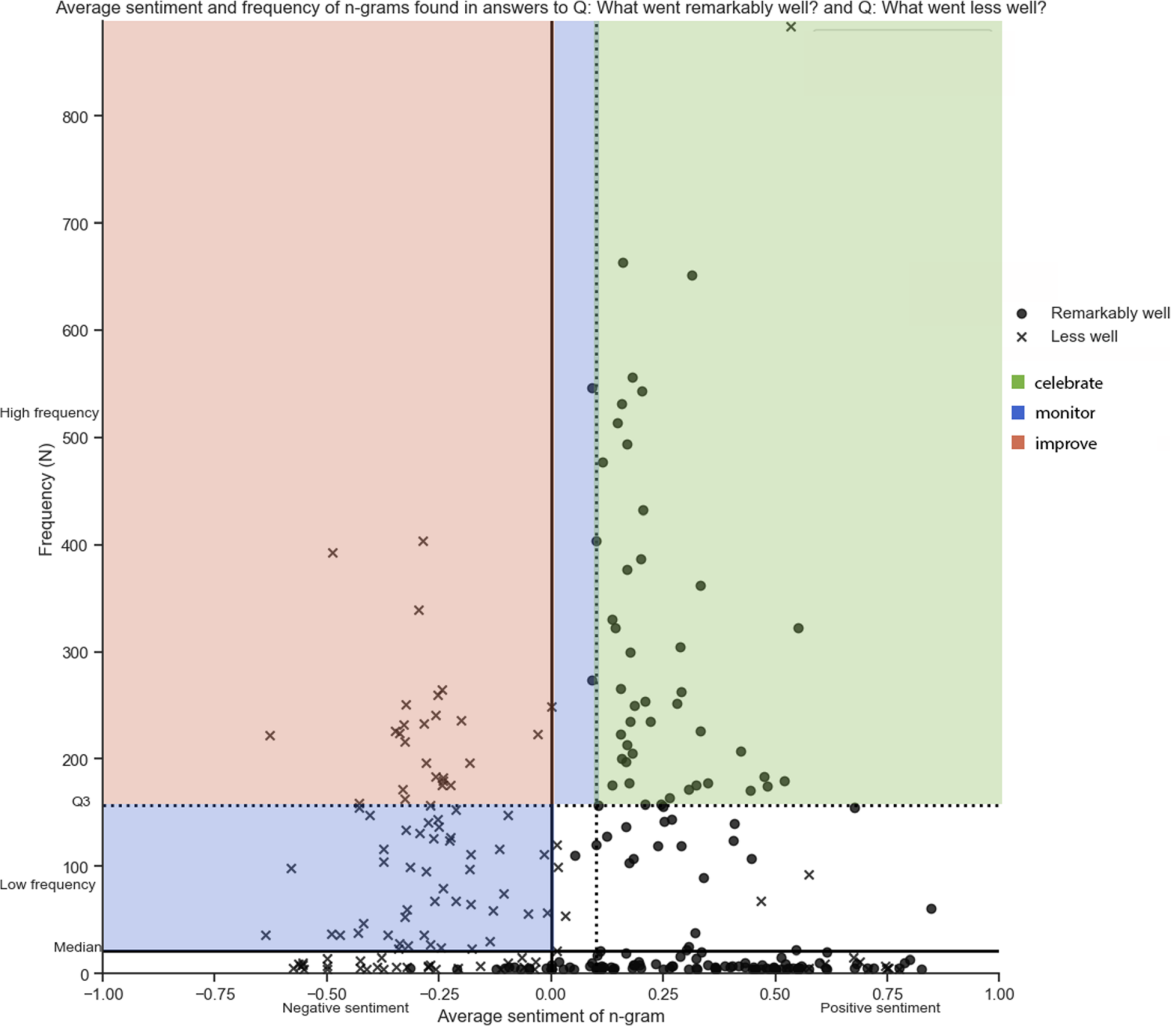
Patient experience priority matrix for hospital 1. Topics to be improved, celebrated, monitored are show in the upper left, upper right and lower left quadrant, respectively

Sentiment and frequency were plotted against each other (Fig. 2). Responses to both questions were plotted in the same matrix using a different visual marker. Topics to celebrate, monitor, and improve on were highlighted. 45 N-grams (15.3%) were highlighted as topics to celebrate, 26 (8.8%) to improve on, 49 (16.7%) to monitor and the other 174 n-grams (59.2%) did not fall into any category. Most topics did not fall into any category because a low number of responses were assigned to it and sentiment scores were neutral. A list of all n-grams and categories can be found in Table 2.

The result of the impact calculation (sentiment times frequency) provides a ranking of n-grams for each category. Table 2 shows the top 5 rankings for each category. The impact score indicates priorities for improvement based on a combination of sentiment and frequency. This results in different priorities than when only frequencies are taken into account. For example, hospital 1 has the n-gram ‘leave early from home’ as a point of improvement in the top 5 priorities, but the frequency is lower than some other topics in that category. It emerges in the top 5 because it has a much lower average sentiment (i.e., more negative experience) score than other n-grams.

### Transferability of the architecture

The architecture of the model used on data from hospital 1, including pre-processing was applied to data of hospital 2, resulting in two different topic models (Table 1). All of the 2608 (100%) submitted surveys had a response to Q1, and 2537 (97.3%) had a response to Q2. Topic modelling for hospital 2 resulted in topics, n-grams, and priorities, as shown in Table 1 and Table 2. The 2 × 2 matrix and a list of topics and n-grams are available in Additional file 1.

Of the 5145 survey responses in total, 4453 survey responses (86.6%) could be assigned a topic. A chi-square goodness-of-fit test was conducted to determine whether an equal number of topics could not be assigned a topic as was the case for hospital 1. The test indicated the percentage of survey responses assigned a topic was comparable to the survey responses from hospital 1 (χ2(2) = 0.083, *p* = .773), thus the topic model was accepted.

For the hospitals, 69.7% of topics were similar. For example, the topics waiting (‘long waiting times’) and communication (‘communication between departments’). For hospital 1, 32.3% of topics were unique. For hospital 2 this was 29.0% These differences include for example the temperature of the room in hospital 1 (‘temperature room low,’ ‘room cold’) and the lack of an emergency department for hospital 2, which was closed in April 2018. The n-grams add context to the one-word topics and distinguish between seemingly similar topics. For example, the topic ‘Room’ for Q2 consists of the n-gram ‘Temperature room low’ for hospital 1, and ‘Lower amount persons room’ for hospital 2.

## Discussion

This study showed NLP techniques can be used to automatically categorize patient experience free-text responses into topics, subtopics (i.e., n-grams), and combine these with sentiment labels. The indicator ‘impact’ was presented in this study to look beyond frequency alone by additionally taking sentiment into account when setting priorities for improvement. Transferability of the model’s architecture was supported as it was updated automatically to capture new, and a comparable number of, topics when used on data from another, general hospital in the same country.

### Automatically defining priorities for improvement

In accordance with previous studies [8, 33, 34] the results show NLP can be used to derive categories from free-text patient experience responses. Most responses (83.6%) to the two questions were categorized into one-word topics. A difference with previous work [5–12] is using unsupervised topic modelling rather than a supervised approach. An advantage of an unsupervised approach is that the topics are an exact representation of the patients’ feedback, without adding interpretation to the data. A supervised approach would not have been able to reveal the n-grams uniquely defined for hospital 2, which represented almost one third of the total number of n-grams. In other words, supervised topic modelling results in topics that are selected in advance, while with unsupervised modelling the resulting topics could be anything.

The use of topic modelling only, as was done in previous patient experience studies [5–12] in one-word categories. N-grams can add interpretability to topic models by adding valuable context to the one-word topics and by distinguish between seemingly similar topics [29]. Even though n-grams provide more information about these categories, this approach also results in a considerable number of n-grams with infrequent words, covering anything from basic hygiene to specific hospital wards. This overload of n-grams can be challenging to interpret and to derive points for improvement. We sought to improve interpretability by only adding n-grams with at least one noun and by creating the new indicator ‘impact’, combining sentiment and frequency. The impact score resulted in different priorities compared to when only frequencies would be taken into account.

### Transferability

Using the same NLP method on a second hospital’s dataset resulted in different topics, n-grams, and priorities even though the dataset was only one-tenth of the size of the primary hospital’s sample. Thereby, this study demonstrates not every hospital would need its team of data scientists to gain access to these methods for local development, but instead, model architectures can be shared. In terms of the identified priorities for improvement, monitoring and celebration, differences and similarities were found between hospitals, showing how the use of transferable architecture still yielded different topic models. However, the acceptance criteria showing the transferability of the method are based on the assumption that the number of found topics is comparable to the number of topics found in the first topic model. A very small dataset or responses addressing similar topics might not yield an acceptable topic model.

### Strengths and limitations

A strength of this study is its data-driven approach to categorize unstructured patient experiences without the need to use predefined categories and thereby limiting human bias. Furthermore, the combination of frequency and sentiment to create a new indicator for prioritization provides new quantitative insight into unstructured textual data. Thirdly, validating the model’s architecture on another hospital’s dataset provides support for the transferability of the method in other patient experience data samples.

A limitation is that part of the sentiment is inherent to the questions asked. Patients responding to ‘What went remarkably well during your stay?’ with one word (e.g., medication) should be assigned a positive sentiment. However, a neutral response to a positive question is marked as neutral. A solution to this problem could be to study the effect of different phrasings (e.g. ‘What was remarkable during your stay?’ without adding ‘well’). Another limitation is that the topic model labels patient responses with one category, though some mention more than one subject in their response. As an example, the response ‘I liked the doctor’s attention as well as how I was treated in the night’ could be labelled as either ‘attention’ or ‘night.’ For these cases, the topic model assigned the most fitting topic to the response. Other studies successfully applied multi-labelling [35], but this not applied in this study because patient responses in the available dataset were relatively short (on average, < 25 words per response). This limitation also applies to the sentiment score. Each free-text response is given one sentiment score irrespective of the number of topics mentioned. This could result in under- or overestimation of a topic’s sentiment.

### Practical implications

The model gives direction for improvement, but still requires a closer look at n-grams by reading the specific responses related to that topic. The method is therefore not suited to replace reading patient responses but can be used to drill down on the enormous amount of responses available so hospitals can select which domains to study in greater depth. Hospitals could use the defined priorities for improvement as a start for in-depth analysis, which is in agreement with the principle that analysing a small number of responses thoroughly is more valuable than a cursory overview of a large number [36] . Another practical implication would be to reduce the number of closed-ended questions in patient experience surveys and analyse whether patients address these points by themselves in open-ended questions, to identify if shorter surveys yield similar results. The model was created with open source software, which means it can be easily shared with other potential users.

### Future directions

Future research is needed to examine the feasibility of the model to guide quality improvement. The described methods should be applied in practice by hospital improvement teams to find if they are actionable and can be used to improve patient experiences. Another potentially valuable direction could be to determine whether this model can be used to combine different types of patient feedback, such as complaints [37]. This results in a richer dataset with a better overview of what patients find most important. A potential solution to the sentiment being present in the stated questions may be to rephrase the questions. For example, the question ‘Please describe your experiences during your stay’ does not include a sentiment in its phrasing and can therefore provide answers which are more appropriate for sentiment analysis.

## Conclusion

This study demonstrated how NLP techniques can be used to automatically categorize responses and define priorities for improvement. The indicator ‘impact’ takes both frequency and sentiment of topics into account to set priorities for improvement. The model’s architecture was shown to be hospital-independent as it was updated automatically to capture a comparable number of topics when used on another hospitals’ dataset. These methods should be considered for future patient experience analyses to make better use of this unstructured but valuable source of data.

## Supplementary information

**Additional file 1.**

## Data Availability

All data generated and/or analysed during the current study are available from the corresponding author on reasonable request.

## Abbreviations

LDA: Latent Dirichlet Allocation
NLP: Natural Language Processing
NMF: Non-negative matrix factorization
