# Supplementary material for: How to automatically turn patient experience free-text responses into actionable insights: a natural language programming (NLP) approach
Source: BMC Med Inform Decis Mak. 2020 May 27;20:97. doi: 10.1186/s12911-020-1104-5 (PMC7251822; doi:10.1186/s12911-020-1104-5)

**Appendix A – Description of n-gram matching procedure**


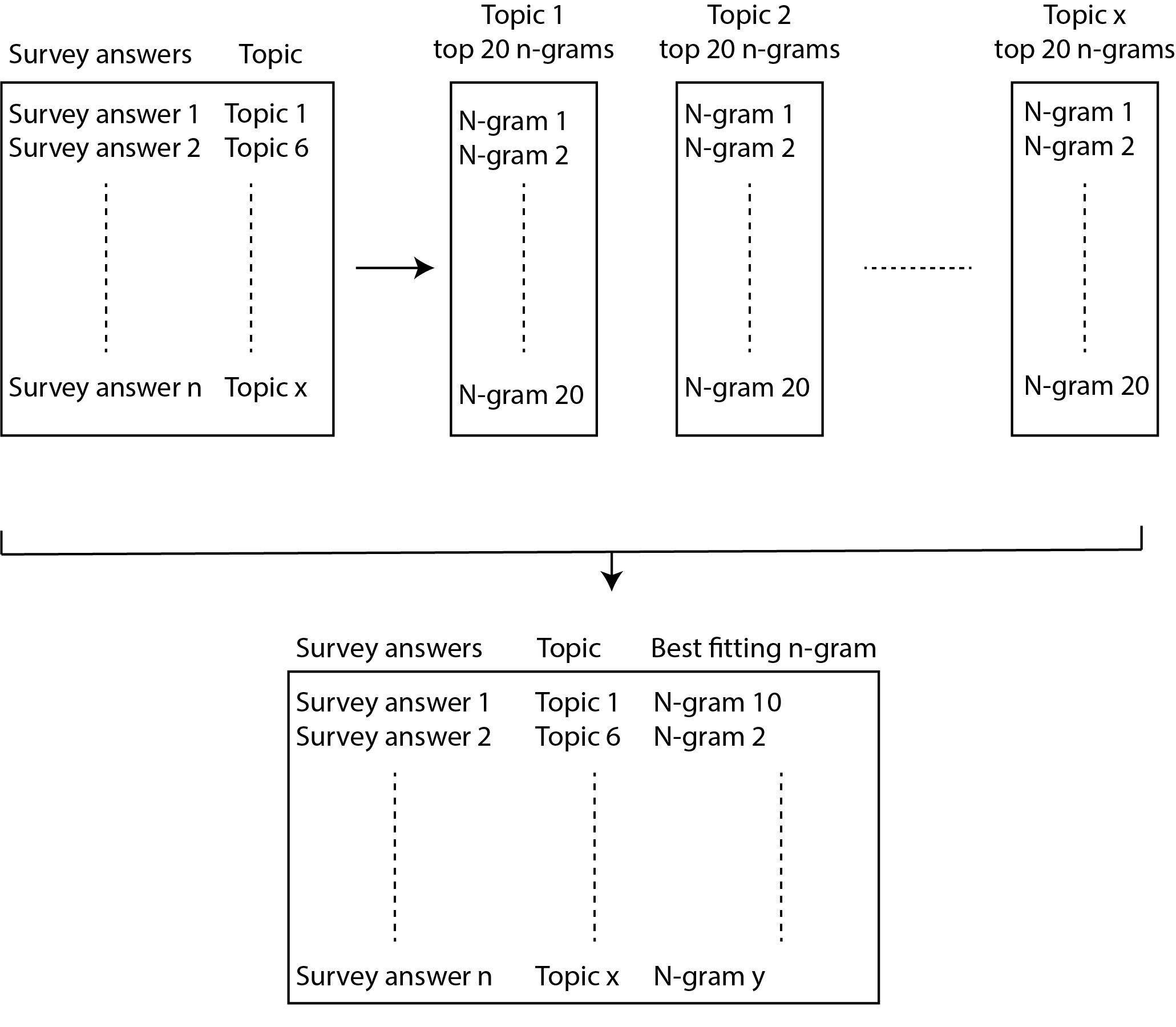


**Appendix B – Impact formula**

The formula to calculate impact is shown below:

$${Scaled frequency}_{i}= \frac{{frequency}_{i}-min(frequency)}{\max\left( frequency \right)-min(frequency)} \times10$$

$${Scaled sentiment}_{i}= {{sentiment}_{i}}^{2} \times10$$

$${Impact}_{i}=\frac{{scaled frequency}_{i} \times{scaled sentiment}_{i}}{10}$$

The sentiment is squared to score extreme sentiments similarly.

**Appendix C – List of n-grams per topic for hospital 1. Prioritized n-grams are highlighted.**

N.B. some n-grams consist of more than n words, and some n-grams are mentioned more than once due to translation from Dutch to English. Duplicate n-grams due to translation (e.g. ‘zeer goed’ and ‘heel goed’ translate to ‘very good’).

^a^ Q1: What went remarkably well during your stay? Q2: What did not go as well during your stay?

| **Hospital 1 – Q1** ^a^ | | **Hospital 1 – Q2** | |
| --- | --- | --- | --- |
| **Topic** | **N-grams** | **Topic** | **N-grams** |
| **1 Friendliness** | Friendliness nursing staff | 1 Nothing | Nothing on this |
|  | Staff very friendly |  | No negative experience |
|  | Staff very friendly |  | No experience |
|  | Staff very friendly |  | No negative points |
|  | Very friendly staff | 2 Everything well | Examples less well |
|  | **Friendly nursing staff** |  | Well during hospitalization |
|  | Friendly involved staff |  | Less well during |
|  | Very friendly staff |  | Hospitalization went well |
|  | Very vriendly staff |  | Good no comments |
| 2 Care | Care during hospitalization | 3 Waiting | Waiting for hour |
|  | Care nursing staff | **4 Waiting time** | Sometimes long waiting |
|  | Good care staff |  | Waiting time CT scan |
|  | Staff good care |  | Very long waiting time |
| **3 Reception** | Reception very good |  | **Long waiting time** |
|  | **Friendly reception department** | **5 No comment** | **No examples did not go well** |
|  | Reception nursing staff | 6 Communication | Communication between departments |
| 4 Surgery | Surgery good |  | Communication between doctors |
|  | Surgery went well |  | Between different departments |
| 5 Medical practice | Care medical staff | 7 No complaints | No complaints |
|  | Medical practice nursing staff |  | No single complaint |
|  | Good medical practice staff |  | Good no complaints |
|  | Staff good medical care | 8 Surgery | Anesthesia eye operation |
| 6 Speed | Speed of hospitalization |  | Waiting room surgery |
| 7 Accompaniment | Good accompaniment explanation |  | Hour before surgery |
|  | Reception accompaniment surgery |  | Insecurity time of surgery |
|  | Accompaniment during hospitalization | **9 Room** | **Temperature room low** |
|  | Accompaniment nursing staff | 10 Not applicable | Question not applicable |
| 8 Attention | Attention nursing staff | 11 Care | Rules medical care |
|  | Good care attention | 12 No example | Give no example |
| 9 Treatment | Treated well nursing staff |  | Mention no example |
|  | Treated well during stay |  | Example of things less well |
|  | Good explanation treatment |  | Example less well |
|  | Fast adequate treatment | 13 Discharge | Information on discharge |
| 10 Hospitalization | Reception during hospitalization | 14 No idea | No idea of care pathway |
|  | Hospitalization went well | **15 Long** | Long waiting surgery |
|  | Went well hospitalization |  | Waiting long surgery |
|  | Good during hospitalization |  | **Long waiting before surgery** |
| 11 Put at ease | Put at ease staff |  | Long wait hospitalization |
|  | Put at ease nurses | 16 Taking blood | Taking blood test |
|  | Nurses put at ease |  | Not so much taking blood |
| 12 Explanation | Clear explanation surgery |  | Less taking blood |
|  | Reception clear explanation | 17 Nothing comes to mind | Nothing comes to mind |
|  | Friendly reception explanation | 18 Treatment | Long wait treatment |
|  | Explanation course of events | 19 Food | Food not good |
| 13 Communication | Communication very good |  | Food not nice |
|  | Good communication nurses | 20 Department | Finding department gave problems |
|  | Communication between doctors | 21 Satisfied | No example satisfied |
|  | Communication nursing staff | 22 Hygiene | Cleaning room better |
|  | Communication between staff | 23 Staff | Halls staff |
| 14 Nurses | Attention doctors nurses |  | Staff complaining |
|  | Accompaniment midwife nurses |  | Saw staff complaining |
|  | Doctors as well as nurses | 24 Nurses | Between doctor and nurse |
|  | Care nursing staff | 25 Time | Long time |
|  | Friendliness doctors nurses | 26 Appointment | No appointment list |
| 15 Time | Speed helped with |  | Different appointments one day |
|  | Help on time | 27 Bed | Bed lies uncomfortable |
|  | Fast accurate help |  | Staying in bed |
|  | Helped emergency department |  | No doctor at bed |
|  | Helpen on the agreed time | 28 Long time | Duration operation |
| 16 Clarity | Staff clear friendly | 29 Questions | Multiple times same questions |
| 17 Kind | Staff kind |  | Same questions were asked |
|  | Staff very kind | 30 Explanation | Explanation on why |
| 18 Preparation | Preparation operation accompaniment | 31 Planning | Not as planned |
|  | Reception preparation surgery | **32 Mistake** | **Went wrong once** |
|  | Hospitalization preparation surgery | 33 Unclear | Unclear signs navigation |
| 19 Personal | Personal attention care |  | Discharge unclear took long |
|  | Personal attention explanation | 34 No suggestions | No suggestions at all |
|  | Personal attention staff | 35 Intervention | Waiting between interventions |
| 20 Nothing | Nothing | 36 IV | Waiting for IV |
| 21 Examination | Accompaniment during examination | 37 Medication | Medication list not known |
|  | Operation examination |  | Check medication at home |
| 22 Intake | Warm welcome intake |  | Medication at home |
| 23 Contact | Contact staff care | **38 Home** | **When could go home** |
|  | Contact nursing staff |  | **Leave early from home** |
| 24 Helpfulness | Staff very friendly helpful | **39 Unclear** | Departments very unclear |
|  | Friendly helpful staff |  | Surgery unclear result |
|  | Staff very helpful |  | **Discharge unclear took long** |
|  | Staff friendly helpful | 40 Medical care | Medical care unsuccessful |
|  | Kind helpful staff | 41 See previous question | See previous question |
|  | Staff very friendly helpful | 42 Phone call | Called an hour |
|  | Staff very kind helpful |  | Wife called surgery |
|  | Staff very helpful | 43 Information | Got information treatment |
| 25 Help | Help very good | 44 Night | Sleep during night hours |
|  | Reception emergency department | 45 Meals | Skipping meals |
|  | Reception first aid | 46 Examination | Present in advance |
|  | Emergency department |  | Operation was done |
|  | Department urgent care | **47 Busy** | Very busy but understand |
|  | Hospitalization first aid |  | **At times very busy** |
| 26 Room | Reception escort room | 48 Pain | Terrible pain |
|  | Nice own room |  | Horrible pain waking up |
| 27 Hospitality | Hospitality reception communication | 49 Emergency department | Waiting emergency department |
| 28 Overall | Information well received |  | Wait emergency department |
| 29 Immediate | Act fast adequate | 50 Pills | Bothering pills |
|  | Acted fast adequate |  | Given at wrong time |
|  | Speed of acting |  | Communication on necessary pills |
| 30 Appointment | All appointments on time | 51 No problem | No problems |
| 31 Welcome | Welcoming reception department | 52 Late | Delayed communication among staff |
| 32 Calmness | Calmness attention department |  | Late no problem |
|  | Surgery went well |  | Hour late communication among |
| 33 Considerate | Very friendly considerate | 53 Aftercare | Late communication between staff |
|  | Friendly considerate staff | 54 Operated | Operated for hours |
|  | Staff friendly considerate | 55 Nothing | Nothing to say |
|  | Caring of nursing staff | 56 Belated | Belated operation |
| **34 Pleasant** | **Pleasant welcome guidance** |  | Hour late |
| 35 Involvement | Staff very involved | 57 Forgotten | Department forgot to get |
|  | Involvement doctors nurses |  | Forget to write medication |
|  | Involvement nursing staff | 58 Attention | Attention questions patient |
| 36 Information | Good information on disease | 59 Perfect | Went perfect |
|  | Information on what will happen |  | Perfect visit |
| 37 Conversation | Conversation good tips | 60 Did not work | Locker did not work |
| 38 Nothing special | Can’t name one thing |  | Did not work during afternoon |
| **39 Sweet** | Sweetness staff smooth treatment | 61 Speed | Fast because busy |
|  | **Staff very kind** |  | Fast surgery not so nice |
| 40 Therapy | Smooth therapy | **62 Cold** | **Room cold** |
|  | Therapy went ok |  | Airconditioning cold |
| 41 Waiting time | Waiting times were ok | 63 On time | Was not helped on time |
| 42 Education | Education what’s happening |  | Only after an hour |
|  | Clarity what’s happening |  | |
|  | Education going to happen |  |  |
|  | Explaining was happening |  |  |
|  | Explain all that happens |  |  |
| 43 Arranged | Intake well arranged |  |  |
|  | Appointments well arranged |  |  |
| 44 Comforting | Room to ask questions |  |  |
|  | Comforting staff |  |  |
|  | I was comforted |  |  |
|  | Well comforted |  |  |
|  | Put at ease |  |  |
| 45 Planning | Appointments well planned |  |  |
|  | Everything as planned |  |  |
|  | All appointments planned |  |  |
| 46 Organized | Well organized |  |  |
| 47 Childbirth | Guidance during childbirth |  |  |
| 48 Expertise | Friendly expert staff |  |  |
|  | Good expertise |  |  |
|  | Expertise nursing staff |  |  |
| 49 Waiting | Long waiting times |  |  |
|  | Explanation while waiting |  |  |
| 50 Staff | Staff kind |  |  |
| 51 Nursing | Before exceptional nursing |  |  |
| 52 Excellent | Excellent complete hospitalization |  |  |
| 53 Taken care of | Great listening |  |  |
|  | Questions got answers |  |  |
|  | Got time and attention |  |  |
|  | Were taken seriously |  |  |
| 54 Co-operation | Collaboration between departments |  |  |
|  | Collaboration between doctors |  |  |
| 55 Approach | Kind words staff |  |  |
| 56 Satisfied | Day appointments right |  |  |
|  | Reception during day |  |  |
|  | Frequent checkups surgery |  |  |
| 58 Well helped | People team treated |  |  |
|  | Respect friendly treated |  |  |
|  | Well treated staff |  |  |
| 59 Everything | Surgery went perfect |  |  |
| 60 Striking | Staff exceptionally good |  |  |
| 61 Food | Good taking care |  |  |
|  | Food drinks |  |  |
|  | Taking care food |  |  |
| 62 Went well | Surgery went well |  |  |
|  | Treatment went well |  |  |
| 63 Capable | Cable nice staff |  |  |
|  | Capable friendly staff |  |  |
|  | Friendly able staff |  |  |
| 64 Total | Extraordinary staff |  |  |

**Appendix D – List of n-grams per question for hospital 2**

Table 4. List of n-grams per topic for hospital 2. Prioritized n-grams are highlighted.

^a^ Q1: What went remarkably well during your stay? Q2: What did not go as well during your stay?

| **Hospital 2 – Q1** | | **Hospital 2 – Q2** | |
| --- | --- | --- | --- |
| **Topic** | **N-grams** | **Topic** | **N-grams** |
| **1 Friendliness** | **Friendliness doctors staff** | 1 Waiting times | Waiting time shorten |
|  | Doctors nursing staff | **2 Patient** | **Time for patient** |
|  | Staff very friendly |  | Take patient seriously |
| **2 Treatment** | **Complete treatment perfect** | 3 Too long | Shorten long waiting times |
|  | Total treatment pleased |  | Lower waiting time |
| **3 Satisfied** | Satisfied complete stay |  | Long waiting hospitalization |
|  | **Very satisfied staff** | 4 Everything good | Care went well |
|  | **Acted quickly with expertise** |  | People were ok |
| 4 Surgery | Surgery went well |  | Good experience |
| 5 Expertise | Friendly expert staff |  | Care very well |
|  | Everyone expertise | **5 Appointment** | Making new appointment |
| 6 Speed | Speed action staff |  | **Waiting time to get appointment** |
| 7 Care | Care of staff | 6 No complaints | No alternative |
| 8 No idea | No complaints treatment |  | No complaints |
|  | No idea |  | Nothing to mention |
|  | No response |  | Compliments hospital |
| **9 Staff** | **Expertise of staff** | 7 **Communication** | **Communication between departments** |
|  | Nice staff |  | Communication between hospitals |
| 10 Nursing | Satisfied with nursing |  | Doctors nurses among each other |
|  | Happy treatment nursing |  | Between departments |
| 11 Nurses | Nurses happy | 8 Satisfied | Satisfied course of events |
| 12 Not applicable | Not applicable |  | Very satisfied |
| 13 Explanation | Explanation advice doctor | 9 Staff | Staff walking around |
|  | Explanation doctor nurses | 10 Room | Lower amount persons room |
|  | Clear explanation doctor | 11 Clarity | Clear information |
| 14 Information | Information friendly |  | Better clearer |
| 15 In general | Staff parking availability |  | Clarity when toilet |
|  | In general satisfied | 12 Food | Food not nice |
| 16 Contact | Contact doctor |  | Plate food not good |
| 17 Aftercare | Care hospital surgery | 13 Departments | First contact department |
|  | Aftercare nurses department | 14 Treatment | Treatment long |
| 18 Tests | Echo tests fine |  | Waiting time treatment long |
|  | Tests done happy | **15 Time** | **Took time** |
|  | Test done protocol |  | Time patient |
|  | Acted follow-up test | 16 Care | Excellent care |
|  | Test results fast |  | Extraordinary care |
| 19 Attention | Friendly attention staff | 17 Parking | Parking difficult |
|  | Honest attention doctor |  | Parking costly |
|  | Care attention department | 18 Change nothing | No change necessary |
| 20 Clarity | Clearly mapping disease |  | Change nothing |
|  | Clear staff | 19 Surgery | Found talk after surgery |
| 21 Dealing with patient | Friendly staff |  | Waiting list surgery |
|  | Make contact with patient |  | Shorter waiting list surgery |
| 22 Total | Total care treatment | 20 Faster | Faster results |
|  | Total treatment |  | Time faster |
| 23 Accompaniment | During day hospitalization | 21 Too busy | Very busy |
| 24 Conversation | Conversation doctor listening | 22 Shorter wait | Shorter waiting times between location |
| 25 Intake | Intake medical treatment | 23 Telephone | Telephone availability better |
|  | Response during intake | 24 Information | Information was missing |
| 26 Time | Taking time to comfort |  | No information beforehand |
|  | Doctor takes his time | 25 Nurses | Idea better sleeping |
| 27 Communication | Communication clear |  | Seeing same nurse again |
| 28 Every part | Happy with every part | 26 Privacy | Room want privacy |
| 29 Food | Food fine |  | No privacy counter |
|  | Food is ok |  | No privacy room |
| 30 Appointment | Arranging appointment |  | Between patient nurse |
|  | Planning easily | **27 Waiting** | Place at home waiting alone |
| 31 Surgeon | Friendly specialist |  | Not nice waiting hall |
|  | Satisfied surgeon |  | **Waiting for results** |
| 32 Skill | Skilled approach doctor |  | **Lower waiting time** |
|  | Doctors skill empathy | 28 English comments | With our stay |
| 33 Specialist | Helped by specialist |  | Positive experience |
| 34 Helpfulness | Hospitality helpfulness | 29 Friendliness | Good experience top |
| 35 Question | No paper questionnaire |  | Friendly service |
|  | Answering questions in peace | 30 Attention | Attention time patient |
|  | Missing questions | 31 Toilet | Rooms toilet cleaner |
|  | Question professionality staff |  | Toilet too small |
|  | Same as previous question |  | Toilet not clean |
| 36 Operation | Hospitalization procedure |  | Toilet shower hall |
| 37 Everyone | Caring staff | 32 Waiting room | Magazines are old |
| 38 *English comments* | Expertise |  | Waiting times waiting room |
|  | Before operation | 33 Expensive | Expensive parking |
| 39 Taking blood | Attention taking blood | 34 Answers | Disease suddenly answers |
|  | Only taking blood |  | People need peace and quiet |
|  | Wrong taking blood |  | Questions not happy |
| 40 Pleasant | Fun during job | 35 Meals | Taste hot meals |
|  | Experience during sad times | **36 Waiting area** | Sometimes busy waiting area |
|  | Staff very pleasant |  | **Long waiting in waiting area** |
|  | Very pleasant nurses | 37 Place to park | No place to park |
| 41 Don’t know | Don’t know |  | Place to park bad coming car |
|  | Not sure | 38 Closer to home | Sending home |
|  | Don’t know looking into |  | Closer to home |
| 42 Doctor | Satisfied doctor | 39 Explanation | Explanation expected waiting time |
|  | Doctor explains |  | Deserves attention |
| 43 Help | Emergency department |  | Better explanation radiologist |
|  | Help nursing floor | **40 Night** | Moment sleep night operation |
| 44 Well helped | Well helped addressed |  | Bad during night |
|  | Speed well helped |  | Working together |
| 45 People | Satisfied people working |  | Patient organizational problem |
| 46 Personal | Respect personal treatment |  | **Only night bad** |
| 47 Accessibility | Accessibility sometimes long wait | 41 Bed | Not bed |
|  | Hospital good accessibility |  | During visit hours |
|  | Hospital central location |  | Bed uncomfortable |
| 48 Procedure | Every part treatment | 42 Tests | Tests must be done |
| 49 Compliments | Sweet friendly compliments |  | Call before test |
|  | Compliments skill doctors | 43 Conversation | Four patients in one room |
| 50 Caring | Caring cleaning lady |  | Waiting conversation doctor |
|  | Caring nursing staff empathy | 44 Coffee | Possibility to get coffee |
| 51 Great | Great |  | Availability of drinks |
|  | Nurses were great | 45 Everything perfect | Everything perfectly fine |
| 52 Content | Everyone very nice |  | Not applicable perfect |
|  | Amazing checkup |  | Perfectly organized |
| 53 Reception | Staff very relaxed | 46 Single room | During conversation doctor |
|  | Tea and coffee perfect |  | Privacy single room |
| 54 Waiting time | Despite waiting time |  | Tv responds remote control neighbor |
|  | Waiting time friendly staff |  | Single room not fine |
| 55 Room | Room to myself | **47 Emergency department** | **No emergency department** |
| 56 Quiet | Quiet atmosphere |  | No one cares |
|  | Quiet hospital plus |  | Emergency department very bad |
|  | Doctors nurses quiet | 48 Fresh air | Fresh air room |
| 57 Diagnosis | Find diagnosis |  | Open small window |
|  | Diagnosis next treatment | 49 Speed | Had to wait arrival |
| 58 No comments | Taken seriously |  | Week home back for operation |
|  | No comments fine |  | Going home fast |
| 59 Preparation | Preparation discharge hospital |  | Very fast home after operation |
|  | Nurses don’t mention | 50 Medication | File a complaint |
|  | |  | Prescription general practitioner |
|  |  |  | No prescription to pharmacy |
|  |  |  | Only after month |

**Appendix E**

Figure 3: Patient experience priority matrix for hospital 2.


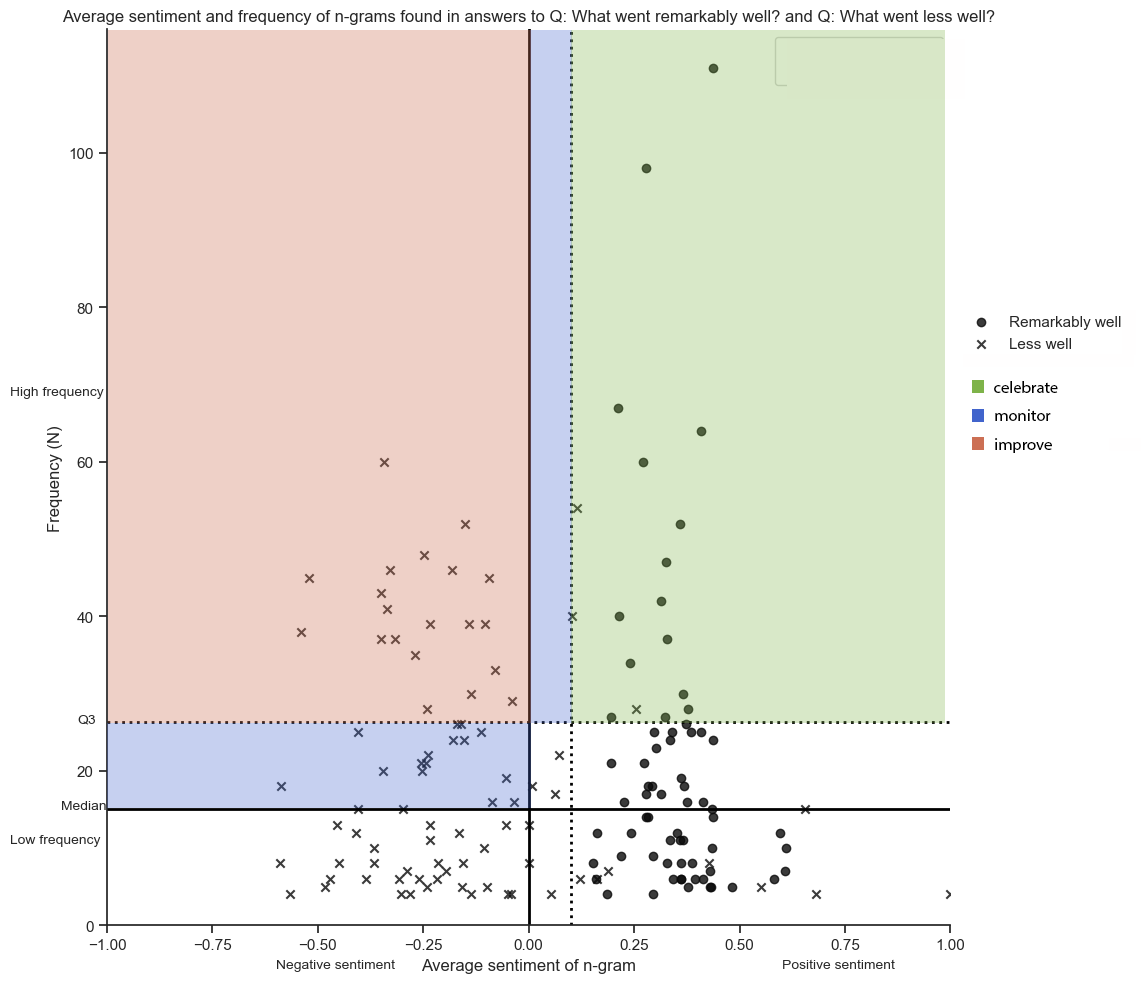

Supplement: Supplementary file 1 — Additional file 1. [file 12911_2020_1104_MOESM1_ESM.docx]
